# Choanal Atresia Repair in Pediatric Patients: Is the Use of Stents Recommended?

**DOI:** 10.7759/cureus.4206

**Published:** 2019-03-08

**Authors:** Abdullah Albdah, Maram Alanbari, Fahad Alwadi

**Affiliations:** 1 Otolaryngology, King Salman Hospital, Riyadh, SAU; 2 Otolaryngology, General Directorate of Health Affairs in Riyadh Region, Riyadh, SAU; 3 Miscellaneous, King Saud Bin Abdulaziz University for Health Sciences, Riyadh, SAU

**Keywords:** choanal atresia, nasal choana, pediatric, stent

## Abstract

Choanal atresia, the obliteration or narrowing of nasal choana, is widely studied across the pediatric and adult population. While unilateral choanal atresia can remain unidentified for several years, bilateral choanal atresia requires immediate intervention, as children are primarily nasal breathers until the initial four months of age. Several surgical methods are reported for repairing choanal atresia in children, and the choice of postoperative management with or without stents is still controversial. In this review, we analyzed several recent studies in which surgery for choanal atresia repair was followed by stent-assisted and/or stentless management. The results of this study are likely to pave the way for a further understanding of the choice of method to increase patency and reduce possible complications.

## Introduction and background

Choanal atresia (CA) is a life-threatening, but relatively uncommon, anomaly involving the congenital obliteration or narrowing of the posterior nasal choana, resulting due to the blocked oronasal membrane in children [1]. The incidence of CA is one in 5-9000 live births [2]. The nasal obstruction in CA can be either bony or membranous; while early reports suggested the bony and membranous obstruction to be 90% and 10%, respectively [3], more recent studies have indicated 70% mixed bony and membranous and 30% pure bony [4].

CA can also be categorized as unilateral, bilateral, or due to other craniofacial abnormalities. Unilateral CA affects only one nostril, usually the right, more - in the ratio of 2:1 [1]. In such cases, the child is often undiagnosed, and the requirement for treatment is realized only when nasal obstruction and unilateral rhinorrhoea is observed. In contrast, bilateral CA can be identified right after birth in most cases, as infants preferably breathe from the nose until four months of age due to the high cervical location of the larynx [5]; therefore, bilateral CA in neonates is considered lethal. Children with craniofacial abnormalities form a very small subgroup of the population and have thick pterygoid lamina [6].

Surgically, CA can be managed with various procedures, and the success is primarily measured based on the “patency” of the choana although several studies have also considered a number of operations, duration of stenting, and so on among other measures. Although the treatment techniques for CA have been extensively reported, there is no consensus on the choice of treatment, as the outcomes are affected by several other conditions, such as feeding patterns and global progress, which, in turn, affect the overall development of the patients. Nevertheless, the various surgical strategies to treat CA include transnasal puncture, transpalatal resection, and endoscopic resection, which can be supported with or without stents [6].

Traditionally, stenting of the opened choana has played a key role in the postoperative management of CA. Park et al. [7] reported that 92 of 95 associates of the American Society of Pediatric Otolaryngology consistently deployed stents. However, in recent years, several studies have suggested that the use of stents is not always required after endoscopic surgery, and their use is still controversial, as cases of stent-related injuries, local infections, inflammation, and necrosis, potentially resulting in permanent septal perforation or cosmetic deformity and ulcerations have been reported [8-9]. However, the potential advantages of using stents include the avoidance of early restenosis, assisting the healing of mucosal flaps, and the prevention of post-surgery edema [10].

The present review provides a comprehensive study of the management of CA with or without stents in the pediatric population, based on the literature, and identifies potential areas for future studies.

## Review

Methods

Papers relating to CA repair in pediatric patients with or without the use of a stent were identified from Google Scholar, PubMed (up to 2019), and the world wide web. The relevant titles and abstracts were searched using the following key terms “choanal atresia,” “stent,” “stentless,” and “pediatric.” Studies pertaining to “adults,” “craniofacial abnormalities,” and the use of “mitomycin C” were excluded. Furthermore, literature references were thoroughly scanned for additional papers, and relevant articles were also studied. The principal search criterion for every article was CA-related outcomes with or without stents in pediatric populations, and relevant articles up to the year 2019 were included in the review.

Results

Several studies have compared the patient outcomes pertaining to the repair of CA in the pediatric population with or without the use of stents. The relevant papers were analyzed and included in the study.

Stent-assisted Repair of Choanal Atresia

Several studies have reported the success of transnasal endoscopic CA repair with the use of stents. Favorable outcomes largely depend on multiple factors, such as the material of the stent, the positioning of the stent, the duration of stenting, and the type of stent. One such study by Nithyasundar & Narayanan [11] described the role of the material of the stent in the success of the surgical outcome. Stents made of soft polyvinyl chloride (Portex, Smiths Medical, Inc., Minnesota, United States) were used in patients aged six days to two years, of whom two had bony CA, one had bony membranous CA, and one had membranous CA. Also, two patients had bilateral CA and two had unilateral. Stents were used in all cases, and the patients were followed up postoperatively for one to two years. The surgery outcomes showed 100% success, and revision surgery was not required in any case. The authors concluded that several factors played a key role in improving the success rate, such as the use of Portex stents, broad-spectrum antibiotics throughout the stenting period, minimum four weeks of stenting, and, thereafter, gentle removal of the stent to prevent trauma and bleeding. Another study by Rodriguez et al. [12] retrospectively evaluated 49 pediatric patients of age three days to 13 years with CA, who were treated with the transnasal microscopic method and a silicone endonasal stent was placed for one to 12 weeks. Of these, 35 patients required revised surgery and nine had complications. Overall, the study found stent-assisted repair to be successful and effective in the pediatric population.

The positive effect of the duration of stenting on surgical outcome was demonstrated in a study by Freitas & Berkowitz [13], who reported stent-assisted transnasal endoscopic repair of CA in 23 neonates of age zero to 32 days. All patients were stented for an average of 75 days, of which 20 were sufficiently followed up. Of these, six required revision surgery, three had one revision procedure, and three had no revision procedure. The study concluded that stenting for a period of three months decreased the chances of early stenosis. In another interesting case, Gupta & Kaur [14] presented the case study of a newborn baby with bilateral CA, who was treated with transnasal endoscopy with stents. The stents were kept for four weeks, and the child was followed up for one year, with no complications.

Similar results were reported by Riepl et al. [15] who treated six patients of bilateral CA of age three days to two months. Transnasal endoscopy was performed, and stents were placed in all the cases for at least six weeks. The study suggested that bilateral stents should be used, particularly in very young patients, to avoid early restenosis. Comparable to the abovementioned study, Romeh & Albirmawy [16] also retrospectively analyzed cases that performed the stent-assisted transnasal endoscopic repair for the management of CA. In total, the cases of 54 children of age seven days to 14 years were analyzed; the duration of stenting was five to seven days to avoid any complications. Thus, a shortened period of stenting was reported to be one of the factors resulting in successful patient outcomes.

Standard endoscopic techniques are typically deployed to manage CA. However, Jones et al. [2] reported an exceptional case of bilateral CA in an infant with CHARGE (coloboma, heart defects, CA, retardation of growth, genital abnormalities, and ear abnormalities) syndrome and difficult anatomy of the skull base. A curved mastoid burr was utilized; stents were placed on Day 1 post-operation and were removed after one week. Breathing and feeding were found normal. After one month, the patient reported increased work of breathing and nasal congestion, which was treated well. After three months, revised choana displayed atretic bone; therefore, treatment with the curved burr was repeated, and the child was successfully treated.

Recently, advancements in the types of stents used have shown favorable outcomes and reduced the otherwise commonly occurring side-effects of using traditional stents. For example, steroid-eluting stents have shown promising results in the post-surgical maintenance of sinus ostia patency and synechiae prevention in adults [17-18]. Bangiyev et al. [19] utilized mometasone furoate steroid-eluting stents (Propel, Intersect ENT, Menlo Park, California, United States) to treat CA in three pediatric patients of age two years, one day, and 16 years, hoping to avoid postsurgical stenosis. The first two cases were identified with bilateral CA whereas the third case exhibited unilateral CA. Case 1 had membranous atresia, Case 2 had bony atresia, whereas Case 3 had mixed type atresia. The stent was successfully deployed in all the cases, and it was found safe and effective, with no restenosis in any patient with 12 months follow-up. Interestingly, the steroid stent was used “off-label,” as it has been Food and Drug Administration (FDA) approved only for the adult population. As a topical nasal spray, the steroid has been FDA approved at 100 μg per day dosage for children of age more than two years; however, it is presently not approved for children of age less than two years. Therefore, the authors recommended further studies on safety profiling and analyzing the long-term effect and efficacy in the pediatric population.

Stentless Repair of Choanal Atresia

Although numerous studies have reported the success of using stents post-surgery in the repair of CA, several studies have also reported favorable outcomes without the use of stents. In a retrospective study, Brihaye et al. [20] comprehensively analyzed the outcomes of surgery for CA without the use of a stent in 36 children, from 1999 to 2015, with 50% unilateral and 50% bilateral cases. Fibrin glue was used to attach the mucosal flaps, and the patients were followed up for six years on average. The authors concluded that surgery was safe for newborns and that restenosis could be minimized by not using the stents and following proper management procedures. Similarly, another successful surgery was documented by Saitabau [21], who presented the case of a 16-year-old girl with bilateral congenital CA of mixed type, who was treated at the Muhimbili National Hospital, Tanzania. Atretic choana was endoscopically treated without stenting under general anesthesia.

The repair of CA by transnasal endoscopy has been widely accepted. Schoem [9] retrospectively reviewed the outcomes without stent in 13 children aged two to 13 days with unilateral and bilateral CA. All the patients were given combinations of oral antibiotics, oral steroids, and topical nasal steroids. The study concluded that repair of CA by transnasal endoscopy is safe and effective without the use of a stent. El-Ahl & El-Anwar [22] also evaluated the stentless repair of CA by the transnasal endoscopic approach. The study involved seven patients of age three-15 days with bilateral CA. The surgery avoided stent-related complications, and the study concluded that the technique was effective, with good patency and faster recovery of the patients. Similarly, El-Anwar et al. [23] also presented a study of 25 patients of age range three to 15 years, in whom bilateral CA was repaired via the stentless transnasal endoscopic approach. During follow-up, wide choana was seen in 18 patients, narrow choana in six, and restenosis in one patient. Overall, the results were found satisfactory without using stents.

The transseptal approach to repair CA without the use of stents was reported by Wormald et al. [24] in 16 pediatric patients and one adult patient. In total, seven cases were bilateral, and 10 were unilateral. The study reported the requirement of postoperation transfusion due to intraoperative bleeding in two neonates and postoperative respiratory complications in two patients. The study recommends following the stentless approach for lasting patency.

Comparative Studies of CA Repair With and Without Stent

Several studies have comprehensively evaluated the effect of stent-assisted and stentless postoperative management by comparing the two in the same patient population. While many studies have reported similar success rates with or without the use of stents, others have reported the advantage of the stentless method over the stent-assisted approach.

Wolf et al. [10] retrospectively reviewed the cases of pediatric patients of age less than 18 years, who underwent endoscopic surgery with or without stents from 2001 to 2012 at their department. The pediatric population treated comprised both unilateral and bilateral CA of mixed and body type and a 100% patency rate was reported in both groups, with no significant differences. The study concluded that outstanding postoperative results could be accomplished both with and without stents. Also, stents were recommended in newborns to prevent restenosis and following lethal complications. However, in children of age more than one year with mild symptoms, stents were not recommended due to the possibility of complications. Similar results were also reported by Moreddu [25] in a review in which stenting was performed in 85 of 114 patients from November 1986 to November 2016. The study concluded that no stent was equally safe, as no significant difference was seen in terms of the number of procedures or patency. However, stent duration (mean 27.7 days) was negatively related to the long-term analysis. In another retrospective study, Kim et al. [26] evaluated the factors influencing the outcomes of surgery on patients aged six days to 28 years. No significant differences in surgical outcomes were seen with or without the use of stents, as the rate of restenosis was 42.9% with stent and 47.4% without a stent. Tomoum et al. [27] reported a comparative evaluation of the endoscopic repair of CA in 72 newborns using a mirrored L-shaped flap without stenting versus stenting without a flap. Patient follow-up revealed higher granulation tissue in patients with a stent (53.3%) than without (28.6%) and 33.33% stenosis in patients with stent and 21.40% in patients without a stent. The study concluded that stentless endoscopic repair with a flap was safe and effective. Another study by Newman et al. [28] compared stent-assisted and stentless management of CA in 42 children of age three days to 15 years. Stents, when used, were kept for 15 days or more; however, the differences were not significant between the groups. The study also suggests the basis of results to be a potential bias, as stents were deployed based on the severity of the disease and, therefore, recommends that stents should be used or not based on the specific case.

The advantage of the stentless method over the stent-based method was reported by Saafan [29] in 20 children, 10 with and 10 without stents. The stent was kept for four weeks, and follow-up for one to two years revealed that choanal narrowing, stenosis, and granular tissue formation were significantly higher in the stent-assisted group, whereas no significant differences were observed in terms of closure. Thus, the study concluded that the use of stents might not be necessary for the repair of bilateral CA. Similar results were reported by Uzomefuna et al. [30] for the stent-assisted repair of CA via transnasal endoscopy in 31 children of age one day to 15 years. The incidence of restenosis was higher in cases with stents; 80% for age less than 10 months and 57% for stents in ages four to six weeks, in comparison to those with no stents, showing 33.3% restenosis, thus concluding that restenosis cases were higher with the use of intranasal stents. Furthermore, Eladl & Khafagy [31] presented a retrospective study of 112 infants of age one to 28 days who underwent endoscopic treatment for the repair of bilateral CA with or without a stent. Restenosis was observed in 42% of patients, and 74.5% of patients in the stent group required a second evaluation in comparison with 20.6% in the non-stent group. The patients had stents for nearly two to six weeks. The study reported that a higher success rate was achieved without the use of stents.

Discussion

Treatment strategies for CA have been described since the mid-nineteenth century; however, the choice of ideal technique is still debated. The present review focused on the stent-assisted and stentless repair of CA in the pediatric population. A wide variation in techniques and outcomes was seen, and several factors were observed to contribute to the success of the surgery. Ideally, the success of the surgery is measured in terms of the higher patency and lower incidence of stenosis, hospital stay, morbidity, and mortality [32]. Several techniques have been reported for the treatment of CA, of which the most commonly used are endoscopic transnasal, transseptal, and transpalatal.

The success of CA depends on the surgery as well as minimal or absent postoperative complications. The choice of using a stent or not has been widely discussed in the literature, and although there appears to be no consensus, certain factors are known to increase the chances of success with minimal complications, such as the choice of the material of stent, the duration of stenting, and patient age. The most commonly observed complications following the surgical correction of CA are stenosis and granulation tissue. While these have been commonly reported with the use of stents, some studies have suggested that the use of soft material stents rather than traditional stents could reduce the complications. For example, Lazar & Younis [33] and Nithyasundar & Narayanan [11] reported exceptional results with the use of Portex polyvinyl chloride stents, whereas Rodriguez et al. [12] reported the use of silicone stents and Bartel [34] suggested the use of a Foley catheter to avoid stent-related complications. In addition, the “off-label” use of drug-eluting stents also gave promising results, though further studies are required to confirm the same [19].

Stent duration and the outcome of the duration of stenting was found to be another factor that affected the successful repair of CA in pediatric patients. The studies’ results were found controversial. Most of the studies reported successful outcomes with longer periods of stenting; Nithyasundar & Narayanan [11] recommended a minimum of four weeks of stenting and a longer duration of stenting with no complication was seen in studies by Rodriguez et al., Freitas & Berkowitz, Gupta & Kaur, and Riepl et al., who deployed the stents for one to 12 weeks, 11 weeks, four weeks, and six weeks, respectively [12-15]. However, some studies also reported the shorter duration of stenting as the possible reason for the successful outcome. The study by Romeh & Albirmawy [16] deployed stents for five to seven days and found favorable outcomes in the patient population. However, some studies have recommended three and six weeks of stenting for unilateral and bilateral CA, respectively [34] although other studies suggest that the duration of stenting should be individualized till the development of mucosa [33].

The stent-associated success of CA repair was also found to vary depending on the age of the patient at the time of the surgery. Wolf et al. [10] reported that the use of stents is the ideal treatment of choice for newborns to avoid restenosis and other complications and for sufficient weight gain before the surgery. They also stated that in children more than one-year-old, with mild symptoms, stents should be avoided due to the possible complications. Thus, while several studies have shown superior results with stentless surgery in comparison with stent-assisted surgery, the reason could be the age of the pediatric population studied. This could be the explanation for the observations by Saafan [29], where the use of a stent in children aged one to five years showed significantly higher complications. However, no such direct evidence exists confirming the proportional risk of stent-assisted surgery with age, and, therefore, the choice of postoperative management for CA repair often depends on the surgeons’ preference [35].

This review had a few limitations. First, we excluded stentless surgery with mitomycin treatment, which could affect the possible success rate observed with stentless management. Second, these observations are not applicable to every case of postoperative management of CA repair due to the possible effect of other previously mentioned factors. Also, this research selectively reviewed the pediatric population only; hence, these results cannot be generalized for the adult population. Third, some articles could have been missed and not included in the study. Fourth, most of the choanal atresia associated with craniofacial anomaly syndrome was not included in this study. Therefore, further studies are required to establish a standard procedure to be followed for the postoperative management of CA repair.

## Conclusions

We believe that the surgical outcomes of CA repair without stents showed fewer complications, thus decreasing the intensity of postoperative management. The stent-assisted procedure could be preferred for younger patients with no complications; however, the use of stents should be evaluated for every case, as several factors affect patency. However, further high-level studies are required to draw clear conclusions.

## References

[1] Anajar S, Hassnaoui J, Rouadi S, Abada R, Roubal M, Mahtar M (2017). A rare case report of bilateral choanal atresia in an adult. Int J Surg Case Rep.

[2] Jones DJ, Vandjelovic ND, Gonik NJ (2019). Novel use of a curved mastoid burr in the management of a difficult case of choanal atresia [In Press]. Otolaryngology Case Reports.

[3] Fraser JS (1910). Congenital atresia of the choanae. Br Med J.

[4] Brown OE, Pownell P, Manning SC (1996). Choanal atresia: a new anatomic classification and clinical management applications. Laryngoscope.

[5] Voegels RL, Chung D, Lessa MM, Lorenzetti FT, Goto EY, Butugan O (2002). Bilateral congenital choanal atresia in a 13-year-old patient. Int J Pediatr Otorhinolaryngol.

[6] Ramsden JD, Campisi P, Forte V (2009). Choanal atresia and choanal stenosis. Otolaryngol Clin North Am.

[7] Park AH, Brockenbrough J, Stankiewicz J (2000). Endoscopic versus traditional approaches to choanal atresia. Otolaryngol Clin North Am.

[8] Van Den Abbeele T, François M, Narcy P (2002). Transnasal endoscopic treatment of choanal atresia without prolonged stenting. Arch Otolaryngol Head Neck Surg.

[9] Schoem SR (2004). Transnasal endoscopic repair of choanal atresia: why stent?. Otolaryngol Head Neck Surg.

[10] Wolf A, Lang‐Loidolt D, Koele W, Wolf G, Thurnher D, Stammberger H, Tomazic PV (2016). Are stents beneficial in endoscopic choanal atresia repair of newborns and children? Case series of 11 patients. Clin Otolaryngol.

[11] Nithyasundar A, Narayanan DS (2016). Choanal atresia: experience with transnasal endoscopic technique. J Pharm Sci Res.

[12] Rodríguez H, Cuestas G, Passali D (2014). A 20-year experience in microsurgical treatment of choanal atresia [Article in English, Spanish]. Acta Otorrinolaringol.

[13] De Freitas RP, Berkowitz RG (2012). Bilateral choanal atresia repair in neonates—a single surgeon experience. Int J Pediatr Otorhinolaryngol.

[14] Gupta M, Kour C (2017). Congenital bilateral choanal atresia: a rare case. J Rare Disor Diagn Therap.

[15] Riepl R, Scheithauer M, Hoffmann TK, Rotter N (2014). Transnasal endoscopic treatment of bilateral choanal atresia in newborns using balloon dilatation: own results and review of literature. Int J Pediatr Otorhinolaryngol.

[16] Romeh HE, Albirmawy OA (2010). A 13-year experience and predictors for success in transnasal endoscopic repair of congenital choanal obliteration. Int J Pediatr Otorhinolaryngol.

[17] Forwith KD, Chandra RK, Yun PT, Miller SK, Jampel HD (2011). ADVANCE: a multisite trial of bioabsorbable steroid‐eluting sinus implants. Laryngoscope.

[18] Murr AH, Smith TL, Hwang PH, Bhattacharyya N, Lanier BJ, Stambaugh JW, Mugglin AS (2011). Safety and efficacy of a novel bioabsorbable, steroid‐eluting sinus stent. Int Forum Allergy Rhinol.

[19] Bangiyev JN, Govil N, Sheyn A, Haupert M, Thottam PJ (2017). Novel application of steroid eluting stents in choanal atresia repair: a case series. Ann Otol Rhinol Laryngol.

[20] Brihaye P, Delpierre I, De Villé A, Johansson AB, Biarent D, Mansbach AL (2017). Comprehensive management of congenital choanal atresia. Int J Pediatr Otorhinolaryngol.

[21] Saitabau Z, Elimath M, Moshi N, Richard E, Ntunaguzi D (2018). Bilateral congenital choanal atresia in a 16-year old girl at Muhimbili National Hospital, Tanzania. Tanzania J Health Res.

[22] El-Ahl MA, El-Anwar MW (2012). Stentless endoscopic transnasal repair of bilateral choanal atresia starting with resection of vomer. Int J Pediatr Otorhinolaryngol.

[23] El-Anwar MW, Nofal AA, El-Ahl MA (2016). Endoscopic repair of bilateral choanal atresia, starting with vomer resection: evaluation study. Am J Rhinol Allergy.

[24] Wormald PJ, Zhao YC, Valdes CJ (2016). The endoscopic transseptal approach for choanal atresia repair. Int Forum Allergy Rhinol.

[25] Moreddu E, Rossi ME, Nicollas R, Triglia JM (2018). Prognostic factors and management of patients with choanal atresia. J Pediatr.

[26] Kim H, Park JH, Chung H, Han DH, Kim DY, Lee CH, Rhee CS (2012). Clinical features and surgical outcomes of congenital choanal atresia: factors influencing success from 20-year review in an institute. Am J Otolaryngol.

[27] Tomoum MO, Askar MH, Mandour MF, Amer MA, Saafan ME (2018). Stentless mirrored L-shaped septonasal flap versus stented flapless technique for endoscopic endonasal repair of bilateral congenital choanal atresia: a prospective randomised controlled study. J Laryngol Otol.

[28] Newman JR, Harmon P, Shirley WP, Hill JS, Woolley AL, Wiatrak BJ (2013). Operative management of choanal atresia: a 15-year experience. JAMA Otolaryngol Head Neck Surg.

[29] Saafan ME (2013). Endoscopic management of congenital bilateral posterior choanal atresia: value of using stents. Eur Arch Otorhinolaryngol.

[30] Uzomefuna V, Glynn F, Al-Omari B, Hone S, Russell J (2012). Transnasal endoscopic repair of choanal atresia in a tertiary care centre: a review of outcomes. Int J Pediatr Otorhinolaryngol.

[31] Eladl HM, Khafagy YW (2016). Endoscopic bilateral congenital choanal atresia repair of 112 cases, evolving concept and technical experience. Int J Pediatr Otorhinolaryngol.

[32] Pirsig W (1986). Surgery of choanal atresia in infants and children: historical notes and updated review. Int J Pediatr Otorhinolaryngol.

[33] Lazar RH, Younis RT (1995). Transnasal repair of choanal atresia using telescopes. Arch Otolaryngol Head Neck Surg.

[34] Bartal M (1988). Improvement stent for use in management of congenital choanal atresia. J Laryngol Otol.

[35] Josephson GD, Vickery CL, Giles WC, Gross CW (1998). Transnasal endoscopic repair of congenital choanal atresia: long-term results. Arch Otolaryngol Head Neck Surg.

